# Comprehensive Medication Management as a Standard of Practice for Managing Uncontrolled Blood Pressure

**DOI:** 10.3389/fmed.2021.693171

**Published:** 2021-08-03

**Authors:** Tony Kuo, Steven Chen, Sang-Mi Oh, Noel C. Barragan, the American Heart Association Western States Chronic Disease Prevention and Management Committee

**Affiliations:** ^1^Department of Family Medicine, David Geffen School of Medicine at University of California, Los Angeles, Los Angeles, CA, United States; ^2^Department of Epidemiology, University of California, Los Angeles Fielding School of Public Health, Los Angeles, Los Angeles, CA, United States; ^3^Population Health Program, University of California, Los Angeles Clinical and Translational Science Institute, Los Angeles, Los Angeles, CA, United States; ^4^Associate Dean for Clinical Affairs, University of Southern California School of Pharmacy, Los Angeles, CA, United States; ^5^Western States, American Heart Association, Burlingame, CA, United States; ^6^Division of Chronic Disease and Injury Prevention, Los Angeles County Department of Public Health, Los Angeles, CA, United States

**Keywords:** blood pressure control, comprehensive medication management, medication adherence, team care, pharmacists

## Introduction

Blood pressure (BP) control is critical for preventing cardiovascular disease. In the United States (U.S.), high BP remains the top modifiable risk factor for conditions such as heart disease and stroke (1). Still, achieving optimal management of BP has been elusive in this country. For example, an analysis of the U.S. National Health and Nutrition Examination Survey dataset by Hales and colleagues shows that while hypertension control has improved overall in the nation, increasing from 28.5 to 48.3% between 1999–2006 and 2007–2014 (2), BP control has yet to meet the American Heart Association's target of 70% (3), suggesting that a more integrated approach to managing uncontrolled hypertension such as Comprehensive Medication Management (CMM) may be needed to close this gap.

CMM is an evidence-based, pharmacist-led clinical service that is designed to ensure the optimal use of medications (1). It has been shown to improve the health outcomes of patients with hypertension and related conditions while simultaneously decreasing the costs associated with their medical care. This care innovation is built on team-based care principles, while facilitating regular one-on-one contact between a pharmacist and a patient. The intervention's primary goal is to foster a longitudinal pharmacist-patient relationship that can be leveraged to problem-solve medication-related problems such as medication therapy appropriateness, safety, cost, and treatment adherence. Pharmacists providing CMM services function as an integral member of the medical team, working closely with physicians, nurses, medical assistants, social workers, and other healthcare professionals and support staff who coordinate whole person care of the patient.

## Emerging Evidence in Support of CMM's Effectiveness

CMM's effectiveness has been demonstrated in a recent Centers for Medicare and Medicaid Services Innovations (CMMI) Health Care Innovation Award project led by the University of Southern California School of Pharmacy and the AltaMed Health Services Corporation (1, 4, 5). The CMMI project showed that providing CMM through integrated clinical pharmacy teams resulted in statistically significant improvements in BP control for participants who were enrolled in the intervention (see Table 1). CMM also produced other improvements in quality measures such as increased medication safety, decreased acute care utilization, and increased patient and physician satisfaction.

**Table 1:** Centers for Medicare and Medicaid Services Innovations Health Care Innovation Award: the Comprehensive Medication Management Project, University of Southern California School of Pharmacy and AltaMed Health Services Corporation, 2012–2015.

**Project description**	**Key findings**
**Background**: In 2012, the University of Southern California School of Pharmacy was awarded a $12 million grant from the Center for Medicare and Medicaid Innovation to implement and integrate pharmacist-led comprehensive medication management (CMM) services in clinics operated by the AltaMed Health Services Corporation. AltaMed is among the largest federally qualified health center systems in Southern California. The goal of the project was to achieve the Centers for Medicare and Medicaid Services' quadruple aims of better care, better health (including better patient experience), lower costs, and better work life for healthcare providers.^a^ **Intervention staffing and delivery sites**: 10 clinical pharmacy teams (clinical pharmacist, clinical pharmacy technician, medical assistant) were assigned to 10 AltaMed clinics, including two *Program of All-Inclusive Care for the Elderly* clinics in Los Angeles and Orange County. At 2 of the clinics, video telehealth capability was available for use. **Evaluation design**: Quasi-experimental design using propensity score matching of intervention participants to comparison participants. **Outcome measures**: National Quality Forum health care quality measures; acute care utilization; patient access to health care services; return on investment; and patient as well as provider satisfaction.	•Over 6,000 patients received CMM from the 10 clinical pharmacy teams during the course of the 3-year project. •More than 67,000 medication-related problems^b^ were identified and resolved by the pharmacy teams during the same 3-year timeframe. •A sub-study early in the intervention showed that among patients with blood pressure above goal at baseline, 87% achieved a systolic blood pressure of <140 mm Hg (average 135.2 mm Hg) and diastolic blood pressure of <90 mm Hg (average 76 mm Hg) at 3 months after start of intervention, representing an overall reduction of 17.8 and 8.0 mm Hg, respectively.

a *The CMMI Comprehensive Medication Management Project has been described elsewhere. Please also see (1, 4, 5)*.

b *Medication-related problems were assigned into four categories: (i) Appropriateness and Effectiveness (e.g., inadequate dosing, untreated medical problem); (ii) Medication Safety (e.g., excessive medication dosing, lack of monitoring); (iii) Non-adherence and Patient-Specific Issues (e.g., underuse of medication or inadequate self-management); (iv) Miscellaneous (e.g., cost/formulary problem, no follow-up)*.

Other studies of pharmacist care (6) have shown similar promise, suggesting that adding pharmacists to the care team can effectively improve management of high risk conditions such as hypertension. Compelling data from a barbershop-based, medication management program in Los Angeles where pharmacists managed uncontrolled BP among black men showed that this variation of CMM was able to achieve a mean reduction of 27.0 mm Hg in systolic blood pressure for those that were enrolled in the intervention; this was a 21.6 mm Hg greater reduction than the mean readings observed in the control group (7). In addition, a recent study from Minnesota found that, as compared to usual care, hypertension patients receiving home BP telemonitoring and CMM through a pharmacist achieved significantly better BP control and a reduction in cardiovascular events by 50% over 5 years (8). The program generated a return on investment of 126% for a net cost savings of ~$1,900 per patient.

## A Call to Action by a Regional American Heart Association Committee

Convened originally in 2016 as the Commission of Blood Pressure Task Force by the Western States of the American Heart Association, the Chronic Disease Prevention and Management Committee's (CDPMC's) two primary goals were: (1) assess the current landscape of innovations and system-level changes that can improve the control of BP in at-risk populations (e.g., older adults, those with modifiable chronic diseases such as diabetes and heart disease, and high risk populations such as Black or Hawaiian Native and other Pacific Islander); and (2) make recommendations regarding which of these innovations can be effectively implemented in both urban and rural communities across the western region of the U.S. – Alaska, Arizona, California, Hawaii, Idaho, Montana, Nevada, Oregon, Utah, and Washington.

Through a systematic process that included an iterative review of the evidence and sequential convenings of its expert membership, the CDPMC concluded in 2019 that the use of CMM should be systematically expanded to support BP control in both clinical and community settings. The recommendation was based on the receptivity of several of the western states' health agencies to scale this model (unpublished internal Committee discussions); a favorable policy environment that has emerged nationally and at the state level to allow for reimbursement of pharmacist-led medication management (thus offering a pathway to sustainability) (9); a growing evidence base suggesting that CMM is effective for controlling BP in high risk populations (1, 6–8); and a strategic calculus that this intervention can help the U.S. healthcare system achieve the quadruple aim of improving care, enhancing patient experience, decreasing costs, and improving the work life of healthcare providers (10).

In making this recommendation, the CDPMC acknowledges there are a number of processes that will be needed to effectively expand CMM across the western U.S. region. These include: (a) better leveraging of health information technology (e.g., clinical decision support, health information exchanges, disease registries, expanded use of telehealth) to streamline and normalize patient encounters using these tools; (b) trainings and strategic convenings of interdisciplinary provider teams to foster team care, allowing all members to practice at the top of their license; (c) greater emphasis on value-based care through use of quality-based performance incentives or system processes that mutually reinforce this paradigm; and (d) policy advocacy at the federal and state level to support increased reimbursement payments for CMM services, including parity in the payments issued for visits using telecommunication vs. those done in person.

CDPMC recognizes the value of CMM and believes that, alongside sensible health policies and support from the medical establishment, implementation of this care innovation could and should be made a common practice to address uncontrolled BP in the U.S., especially for underserved communities where physician access is limited.

## Discussion

The emergence and evolution of CMM in clinical practice offers the U.S. healthcare system an unprecedented opportunity to close the gap in BP control. CMM's versatility and promising impact on healthcare quality across multiple settings—e.g., clinical, community, urban, rural—have persuaded a number of state and local practitioners and policymakers to act. For example, optimizing the workforce and leveraging existing infrastructure to support this care innovation represent priority actions for many western states in the U.S. In states like California, legislative actions such as Senate Bill 493 (9) have expanded the scope of pharmacist practice in anticipation of the need for a better trained workforce that can administer CMM programming. Moreover, in many states, the structure necessary to make this model more common place and accessible may already exist – i.e., an expansive network of highly accessible pharmacies and pharmacy professionals who provide culturally sensitive care. Based on data from the California Department of Consumer Affairs, Los Angeles County pharmacies and pharmacy professionals by zip code are broadly distributed across the county including in rural areas of the jurisdiction (see Supplementary Figure 1).

The present article serves as CDPMC's statement and support of CMM. As a comprehensive team care strategy, this program intervention has the potential to be an effective model for ensuring optimal results from medication therapies and achieving desirable health outcomes while reducing total healthcare costs, especially for patients at highest risk of hospitalization and death from poorly controlled BP and conditions that may heighten this risk (11–14). In promoting this care innovation, the Committee believes that CMM should be a standard of practice for managing uncontrolled BP.

## Author Contributions

TK and SC are the co-chairs of the American Heart Association (AHA) Western States Chronic Disease Prevention and Management Committee. They conceptualized the framework used by the Committee in its assessment and proceedings on comprehensive medication management. S-MO coordinated AHA's effort while NB coordinated the local health department's technical support of this project. All four main authors and co-authors from the Committee as listed in the Committee Members section contributed to the synthesis and development of the overall manuscript. TK wrote the initial draft of the opinion piece. Other Committee members listed as co-authors provided input and expertise to support this work. All authors contributed to its writing.

## Committee Members

Committee contributors to this article include the following: David C. Dugdale, MD, FACP; Teresa Hodgkins, PharmD, BCACP; Charles Magruder, MD; Peter Mann-King, HCPM; Patricia Kim Phuong Nguyen, MD; Nathan D. Wong, PhD, FACC, FAHA; Andrew Terranella, MD, MPH; Joy L. Meier, PharmD, PA, BCACP; and Gina Featherstone.

## Conflict of Interest

The authors declare that the research was conducted in the absence of any commercial or financial relationships that could be construed as a potential conflict of interest.

## Publisher's Note

All claims expressed in this article are solely those of the authors and do not necessarily represent those of their affiliated organizations, or those of the publisher, the editors and the reviewers. Any product that may be evaluated in this article, or claim that may be made by its manufacturer, is not guaranteed or endorsed by the publisher.
