# Supplementary material for: Comprehensive Medication Management as a Standard of Practice for Managing Uncontrolled Blood Pressure
Source: Front Med (Lausanne). 2021 Aug 3;8:693171. doi: 10.3389/fmed.2021.693171 (PMC8369150; doi:10.3389/fmed.2021.693171)

Supplemental Figure 1. Maps Showing the Locations of Retail Pharmacies and Pharmacy Professionals by Zip Code in Los Angeles County.

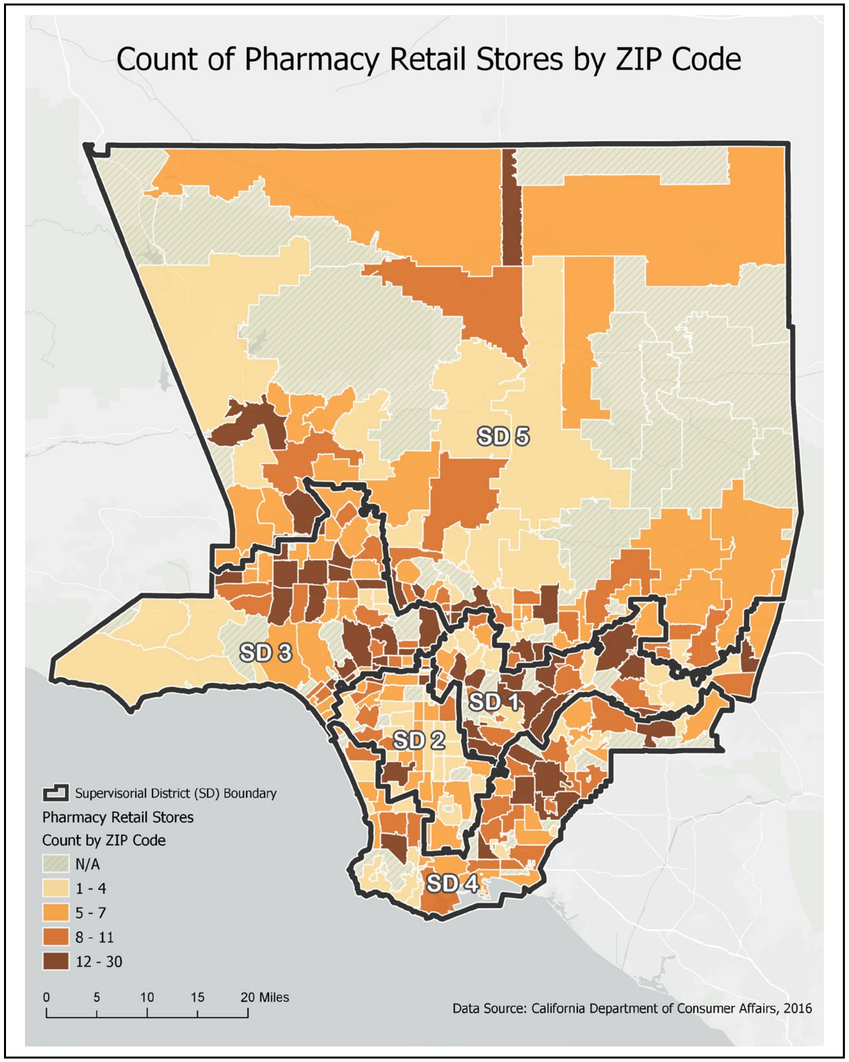

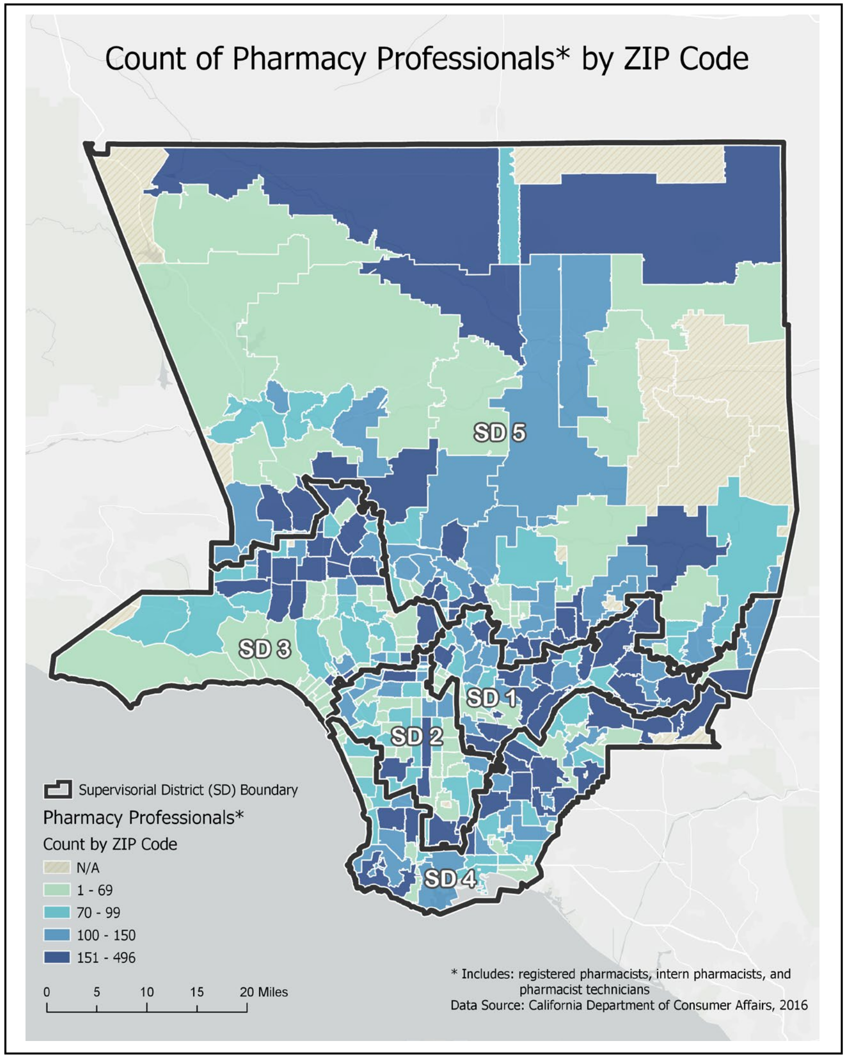

Supplement: Supplementary file 1 [file Image_1.pdf]
